# Identifying Barriers and Facilitators to Recruiting and Retaining Underrepresented Groups in Dementia Prevention Research: Insights from a Scoping Review and Dutch Focus Groups

**DOI:** 10.1002/alz70860_104813

**Published:** 2025-12-23

**Authors:** Awaale Fuad Rirash, Renelle Bourdage, Sanne Franzen, Marieke P Hoevenaar‐Blom, Leonie N.C. Visser, Esther van den Berg, Janne M. Papma

**Affiliations:** ^1^ Erasmus Medical Center, Rotterdam, South Holland, Netherlands; ^2^ Erasmus University Medical Center, Rotterdam, South Holland, Netherlands; ^3^ Erasmus MC ‐ University Medical Center, Rotterdam, South Holland, Netherlands; ^4^ Amsterdam UMC, location AMC, Amsterdam, Netherlands; ^5^ Academic Medical Center/University of Amsterdam, Amsterdam, Netherlands; ^6^ Department of Neurology and Alzheimer Center Erasmus MC, Erasmus MC University Medical Center, Rotterdam, the Netherlands, Rotterdam, Netherlands; ^7^ Alzheimer Center Erasmus MC, Rotterdam, South Holland, Netherlands

## Abstract

**Background:**

Dementia prevention studies in the Netherlands have predominantly targeted affluent native Dutch. This is problematic, since underrepresented populations—often characterized by migration backgrounds, low SES, and low health literacy—face an increased risk of developing dementia. We need more insight into factors that hinder and/or support inclusive prevention research. This study aimed to identify barriers and facilitators for recruiting, engaging, and retaining (RER) underrepresented populations in dementia prevention research through a review of international literature and focus groups considering the Dutch context.

**Methods:**

A scoping review was conducted following the PRISMA‐ScR protocol. We searched for prevention studies and reviews on barriers and facilitators in Medline, Embase, WoS Core Collection, CINAHL, and Cochrane Library, without restrictions. Articles were screened at title/abstract and full‐text levels by two independent reviewers using Covidence; conflicts were resolved by consensus. Two focus groups were conducted with researchers from five Dutch cohort studies, including the HELIUS study, which has been notably successful in recruiting a culturally diverse cohort.

**Results:**

The search yielded 1955 unique articles, resulting in 60 articles selected for data extraction, including 45 dementia prevention trials and 15 reviews on RER in dementia prevention research. All but one of the included articles originate from the U.S. Review and focus groups results were mapped in an infographic, aimed at informing researchers and policy makers. Preliminary synthesis suggests barriers and facilitators across various domains, including mistrust, logistics, a one‐size‐fits‐all approach, personal contact, and sustained interaction. However, key persons, a common facilitator in the review data, was suggested as both barrier and facilitator during the focus groups. Researchers underlined recognizing the importance of within group diversity, as key persons from one subgroup may not represent or engage other subgroups.

**Conclusions:**

The scoping review provides an overview of relevant barriers and facilitators for recruiting and engaging a cognitively healthy diverse study population in the context of dementia prevention research. Results from the focus groups help in understanding factors influencing RER in the Dutch context. Addressing these factors in different cultural settings is essential for developing effective prevention strategies tailored to country‐specific barriers and facilitators, ultimately improving health equity.

